# The Brunswik Lens Model: a theoretical framework for advancing understanding of deceptive communication in autism

**DOI:** 10.3389/fpsyg.2024.1388726

**Published:** 2024-07-11

**Authors:** Tiegan Blackhurst, Lara Warmelink, Amanda Roestorf, Calum Hartley

**Affiliations:** ^1^Department of Psychology, Lancaster University, Lancaster, United Kingdom; ^2^Autistica UK Registered Charity, London, United Kingdom

**Keywords:** autism, deception, perception, detection, frequency

## Abstract

Due to differences in social communication and cognitive functioning, autistic adults may have greater difficulty engaging in and detecting deception compared to neurotypical adults. Consequently, autistic adults may experience strained social relationships or face increased risk of victimization. It is therefore crucial that research investigates the psychological mechanisms that are responsible for autistic adults’ difficulties in the deception process in order to inform interventions required to reduce risk. However, weaknesses of extant research exploring deception in autism include a heavy focus on children and limited theoretical exploration of underlying psychological mechanisms. To address these weaknesses, this review aims to introduce a system-level theoretical framework to the study of deception in autistic adulthood: The Brunswik Lens Model of Deception. Here, we provide a comprehensive account of how autism may influence all processes involved in deception, including: *Choosing to Lie (1), Producing Deception Cues (2), Perceiving Deception Cues (3)*, and *Making the Veracity Decision (4).* This review also offers evidence-based, theoretical predictions and testable hypotheses concerning how autistic and neurotypical adults’ behavior may differ at each stage in the deception process. The call to organize future research in relation to a joint theoretical perspective will encourage the field to make substantive, theoretically motivated progress toward the development of a comprehensive model of deception in autistic adulthood. Moreover, the utilization of the Brunswik Lens Model of Deception in future autism research may assist in the development of interventions to help protect autistic adults against manipulation and victimization.

## Key Recommendations

- Investigate how autistic adults engage in *all four* stages of the Brunswik Lens Model to create a comprehensive model of deception in autistic adulthood and inform evidenced-based interventions and educational programs.
- Research the *truthful and deceptive* behavior of autistic adults to educate the general population and legal professionals on the natural characteristics of autism vs. potential deceptive behavior to reduce the likelihood of erroneous veracity decisions.
- *Work collaboratively* with the autistic community, ensuring autistic adults are invited to provide input relating to research design and participation in deception research.
- Design *naturalistic experiments* in which autistic participants must lie face-to-face to generate realistic data representing everyday social situations.

## Introduction

The ability to deceive and detect deception allows individuals to navigate social contexts, maintain social relationships, and protect oneself against manipulation (Vrij, 2008; Choshen-Hillel et al., 2020). Here, we refer to deception in line with Vrij’s (2008) definition of “a successful or unsuccessful deliberate attempt, without forewarning, to create in another a belief which the communicator considers to be untrue” (p. 15). Due to the inherently social nature of deception and supposed perspective-taking requirements, it is widely believed that autistic individuals who experience difficulties with social understanding may struggle to tell and detect lies (Williams et al., 2018). Autism spectrum disorder (ASD) is a heterogenous neurodevelopmental condition, classified by varying levels of complications with social communication and social interaction (American Psychiatric Association, 2013). Each autistic individual experiences a unique profile of varying strengths and weaknesses related to their adaptive functioning and cognitive development (e.g., language, executive functioning, and perceptual sensitivity; American Psychiatric Association, 2013; Meilleur et al., 2015). Crucially, difficulties deceiving others may strain autistic adults’ social relationships (Henderson et al., 2023) and difficulties detecting deception may increase their risk of falling victim to crimes, such as fraud (Action Fraud, 2019). To note, throughout this review we use identity-first language (e.g., autistic adults) as this is believed to be the preferred terminology among the majority of the autistic community (Kenny et al., 2016; Bradshaw et al., 2021).

Existing research investigating how autistic adults deceive has yet to examine differences in underlying psychological mechanisms and lacks a robust theoretical framework of deception. This is problematic as theoretical understanding of how deception in autism differs, both in terms of individual mechanisms and as an integrated system, is necessary to inform effective interventions that can improve outcomes for autistic adults (e.g., anti-victimization programs to develop personal safety skills and reduce the likelihood of manipulation via deceit; National Institute for Health Care and Excellence, 2021). Therefore, the objective of this paper is to introduce a system-level theoretical framework: the Brunswik Lens Model of Deception (Hartwig and Bond, 2011) to the study of deception in autistic adulthood. This paper will discuss how this model of deception can provide theoretical direction for research investigating how autistic adults produce, perceive, and detect deception, highlighting psychological mechanisms underpinning each inter-related deception process. By adopting this model, the field can make substantive, rapid, and theoretically motivated progress toward attaining a comprehensive understanding of deception in autism.

In 2022, Bagnall et al. conducted a comprehensive synthesis of literature investigating deception in autistic children and adults. Following a scoping review methodology, they identified 28 relevant studies employing naturalistic (e.g., lying face-to-face) and gameplay deception methods (e.g., lying in computerized games). Bagnall et al. (2022) classified these studies into three main themes: (1) Deception ability and prevalence (including gameplay and naturalistic deception), (2) Psychological correlates of deception [e.g., intellectual ability, Theory of Mind (ToM), Executive Functioning (EF); a set of cognitive processes necessary for cognitive control of goal-directed behavior; Suchy, 2015], and (3) Social learning (including training and social contexts). On the balance of evidence, they suggested that autistic individuals find it more difficult than non-autistic individuals to deceive and that autistic adults may deceive others using different psychological mechanisms. For example, it is speculated that in comparison to neurotypical individuals, autistic individuals may utilize socio-cognitive strategies to counteract their difficulties with ToM (e.g., problems with perspective-taking and understanding others’ thoughts and emotions). They may employ working memory and social learning to connect past behaviors and contexts to interpret deceptive behaviors (Livingston et al., 2019a,b; Bagnall et al., 2023). However, as some autistic adults are known to experience difficulties with their working memory (Hill, 2004) and have marked difficulties with social learning (American Psychiatric Association, 2013), such theoretical claims need to be directly investigated before we can draw conclusions about autistic adults’ use of socio-cognitive strategies during deceit.

Bagnall et al. (2022) speculated that deception abilities will vary between autistic individuals, and proposed that for some autistic individuals their deception abilities may be delayed and develop later in life (i.e., when they reach adolescence or adulthood). This developmental trajectory would differ from that observed in neurotypical individuals, whereby lying proficiency generally improves during childhood (age 6–8 years), peaks around early adolescence (age 13–15), and begins to decline from young adulthood (from 16 to 18+) (Debey et al., 2015; Glätzle-Rützler and Lergetporer, 2015). However, as the mean age of participants across all studies included in Bagnall et al.’s review was just 12.86 years, and only 4 studies focused exclusively on adults, further research is required to elucidate lifespan differences in the autistic developmental trajectory for deception.

Moreover, Bagnall et al. (2022) did not identify any studies investigating how autistic adults produce deceptive behavior. The lack of evidence concerning the appearance of deception in adulthood is problematic because natural autistic behaviors, such as gaze-aversion and fidgeting, may be misconstrued by neurotypical observers as stereotypical indicators of deceit (Logos et al., 2021). Such erroneous judgments could potentially have life-changing consequences if an autistic adult’s veracity is questioned in courtrooms or suspect interviews (Blackhurst et al., 2022; Lim et al., 2022). Given these risks, paired with the increased social complexity of deception in adulthood (compared to adolescence and childhood; Walczyk and Fargerson, 2019), it is vital that research directly investigates how autistic adults produce and detect deceptive behavior.

Many existing deception studies describe autistic behavior without directly testing psychological explanations for observed differences in comparison with neurotypical individuals (e.g., executive functioning or ToM). However, understanding how cognitive mechanisms underpinning deceptive communication differ between autistic and neurotypical adults is necessary to inform the design of effective interventions targeting causal factors. Furthermore, as existing research has not been informed by a comprehensive model of deception, there are significant gaps in knowledge. For example, we do not yet know what deception cues autistic adults display or whether autistic adults perceive deception cues similarly to neurotypical adults. Additionally, to our knowledge, just one study to date has investigated deception detection in autistic adults (Williams et al., 2018), reporting reduced accuracy in comparison with neurotypical adults. If autistic adults are particularly vulnerable to deceit, this may increase their susceptibly to crimes involving manipulation or coercion. Providing a pathway to address the limitations of extant knowledge, this paper highlights the utility of working from a joint theoretical perspective provided by the Brunswik Lens Model of Deception (Hartwig and Bond, 2011). This model represents the most unified model of deception; other models are only concerned with the production of deceptive beliefs (e.g., Information-Theoretic Model; Kopp et al., 2018) or appearance of liars’ behavior (e.g., Four Factor Model; Zuckerman et al., 1981). Comparatively, the Brunswik Lens model offers explanations for the production, perception, and detection of deceptive communication, thereby providing a comprehensive account of all processes involved in deception as a system.

In the following sections we review the Brunswik Lens model and offer evidence-based theoretical predictions concerning how autistic and neurotypical adults’ behavior may differ at each step in the deception process, providing testable hypotheses for future research to examine. Where appropriate, we provide alternative theory-driven hypotheses regarding how autism may potentially influence stages of deception both positively and negatively. It is important for researchers to explore the strengths of neurodiversity, as focusing solely on the limitations that autistic adults face during deceptive-decision making could result in personal and/or social harm to the autistic community (Robertson, 2010). Moreover, acknowledging the potential strengths of autistic adults reflects the population’s diversity and is necessary to advance scientific progress toward achieving a comprehensive understanding of deception in autism (Dinishak, 2022).

## The Brunswik Lens model

**Figure 1:**
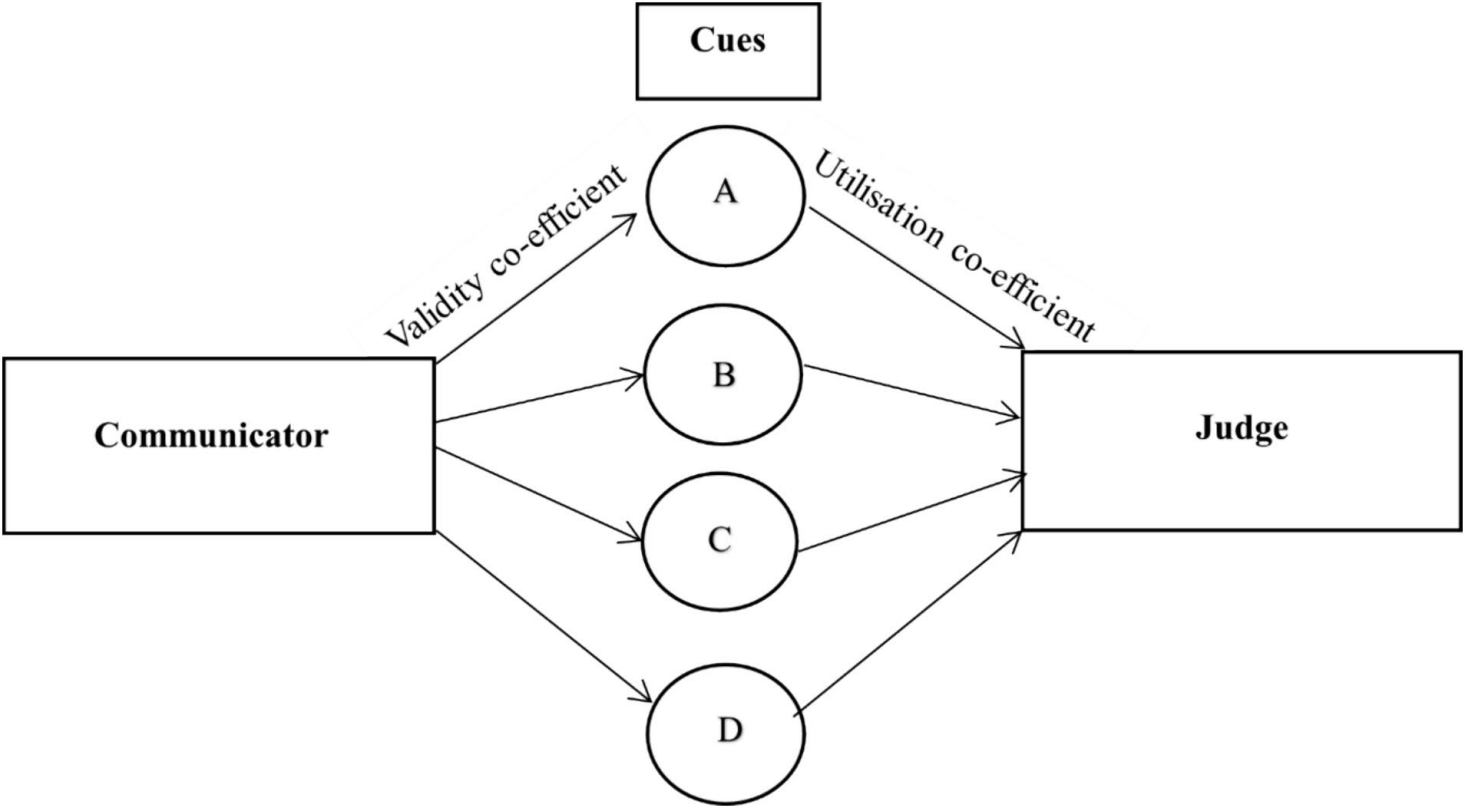
Hartwig and Bond’s (2011) lens model approach to deception detection.

The Brunswik Lens Model (Brunswik, 1952) is a conceptual framework originally designed to study predictions of outcomes probabilistically related to cues (e.g., doctors making predictions regarding a patient’s health based on their symptoms; Karelaia and Hogarth, 2008). The model assumes that humans exist in uncertain environments and so their inferences about the environment rely on probabilistic data (e.g., patient has X and Y symptoms and, while they could relate to different conditions, the doctor will consider the most plausible explanation; Brunswik, 1952; Hammond, 1996). In the model, judgments are based on a range of cues with different ecological validities. Here, ecological validity refers to the correlation between the outcome and the cue (e.g., correlation between a patient with symptom X being diagnosed with condition Z; Hursch et al., 1964). Cues differ in terms of their use, with cue utilization signifying the strength of the association between the cue and the inference drawn by the perceiver (Hartwig and Bond, 2011). The accuracy of the perceiver’s judgment can be calculated by analysing the correlation between the inference drawn from cues and the outcome (e.g., running biological tests to confirm whether the patient has condition Z, as predicted by their doctor based on the presence of symptoms X and Y). Hartwig and Bond (2011) were the first to apply the Brunswik Lens Model to deception (see Figure 1).

The communicator (appearing to the left of Figure 1) can choose to tell the truth or lie—a decision that is signaled by behavioral cues. The validity co-efficients stemming from the communicator to each cue indicate the direction and strength of the relationship between the communicator’s deceptiveness and that cue. To detect deception, a judge (the individual considering whether the communicator is lying) will attend to each cue, inferring deception from certain cues and honesty from others (this process of decoding deception appears to the right of Figure 1). For example, a lying communicator may attempt to make more eye-contact with a judge to appear credible (increased eye-contact is the cue to deceit). Then, the judge will notice this behavior and consider whether it indicates that the communicator is lying or telling the truth. As deliberate attempts to maintain eye-contact may seem suspicious, the judge may decide this behavior is indicative of deceit and judge the communicator to be lying. The lines stretching from the cues to the judge possess a utilization co-efficient representing the strength and direction of the relationship between a cue and whether this leads the judge to infer deceit (Hartwig and Bond, 2011). It is important to note that a judge may use a cue with a validity co-efficient of 0, as not all behaviors are cues to deceit (e.g., averted eye-gaze is a stereotypical deception cue, but is an objectively unreliable indicator of deceit and may therefore have a validity co-efficient of 0; DePaulo et al., 2003). To summarize, Hartwig and Bond’s account of deception can be condensed into four key steps:

1. The communicator chooses whether to lie or tell the truth.
2. The communicator displays certain cues to deception and honesty.
3. A judge perceives cues to deception and honesty.
4. The judge utilizes cues (alongside other information) to make a veracity judgment.

Drawing on existing knowledge of autism, we will apply the Brunswik Lens Model of Deception to autistic adults.

## Step 1: choosing to lie

Step 1 of the Brunswik Lens Model of Deception (Hartwig and Bond, 2011) states that an individual must make an active, conscious choice to inhibit the truth and tell a lie. According to Bok’s (1999) veracity principle, people choose not to lie when telling the truth is most effective for goal attainment. However, if telling the truth presents a barrier to goal attainment when lying does not, people may choose to deceive. While people lie to achieve goals and obtain positive outcomes, Regulatory Focus Theory (Higgins, 2012) proposes that people also lie to foster safety, elude danger, and avoid negative consequences. Individuals may choose to tell prosocial lies, which are socially acceptable and expected in certain situations, more frequently than antisocial lies which are deemed selfish and immoral (Lupoli et al., 2017). Prosocial lies rarely carry severe consequences whereas the risk associated with telling antisocial lies may serve as a deterrent (Wiltermuth et al., 2015). Research suggests that neurotypical adults lie at least once or twice per day on average (DePaulo et al., 1996), although there are large individual differences in lying frequency in the population (Serota and Levine, 2015).

It has been speculated that lying frequency may be reduced in the autistic population due to differences in psychological functioning (Baron-Cohen, 2008). Theory of Mind (ToM) refers to the understanding that people have mental states that can differ from reality (Happé et al., 2017). Awareness of others’ mental states is considered to be a prerequisite for deception as one must consider what their communication partner knows to be true to ensure they do not contradict themselves (Lee, 2013). It is well-documented that many autistic adults have difficulty understanding the mental states of others, although their lower performance on ToM assessments may sometimes be exacerbated by how stimuli and tasks are presented (Andreou and Skrimpa, 2020). Consequently, autistic adults may lie less frequently if they are unable to anticipate their communication partner’s perspective. For example, autistic adults may fail to identify situations in which telling a prosocial lie may be required to maintain social relationships and/or avoid harming others (e.g., answering questions such as “Do you like my new hair style?” with “Yes, it’s lovely” to avoid hurting a friend’s feelings, even if a truthful answer would be “No, I do not like it”). Difficulties representing and understanding another’s perspective may reduce lie frequency within both autistic-neurotypical and autistic-autistic communication. Alternatively, autistic adults may choose to lie as frequently as neurotypical adults, but may find it more difficult to successfully implant false beliefs due to their inaccurate perception of others’ perspectives.

Finally, it is important to recognize that communication between neurotypical and autistic individuals may break down in reciprocity and mutual understanding due to their differing experiences of the world (“the double empathy problem,” Milton, 2012, 2018). Difficulties understanding the intentions and mental states of others within social interactions may therefore occur in both directions, with the ‘double empathy problem’ influencing how likely neurotypical adults are to interact with, and lie to, autistic adults. Differences in experiences at both macro (lifespan/development) and micro (social relationships) levels (Milton et al., 2022) are believed to create a barrier preventing autistic and neurotypical adults from learning about each other’s social behavior (including how to deceive) and social cues (including deception cues). Also, in terms of statistical frequency, it is likely that most neurotypical adults will have significantly less experience interacting socially with autistic individuals compared to other neurotypical individuals (Milton et al., 2022). This lack of exposure and connection between autistic and neurotypical adults may present a barrier to understanding each other’s mental states and social behaviors. As such, lying frequency may potentially be greater between neurotypical-neurotypical adult interactions than neurotypical-autistic interactions due to differences in ease of perspective taking and shared social experiences (Lee, 2013).

While some neurotypical adults find lying stressful, autobiographical reports suggest that negative emotional consequences associated with choosing to lie may be exacerbated in autistic adults. For example, Grandin (2006, p. 156) states that “lying is very anxiety-provoking for me” and Birch (2003, p. 121) states that they “found lies very painful.” Experiencing adverse emotional reactions when lying may discourage some autistic adults from choosing to deceive as frequently as neurotypical adults. However, it is important to acknowledge that a small number of anecdotal reports may not be representative of the broader population. The influence of emotion on one’s inclination to lie offers an alternative explanation for potential differences in lie-frequency between autistic and neurotypical adults that extends beyond ToM, with further research required to advance this prospective explanation.

Limited existing evidence suggests that autistic children lie less frequently than neurotypical children (Talwar et al., 2012). However, there is a notable gap in the empirical literature directly examining lying frequency in autistic adults. In 1996, Yirmiya et al. conducted the first study examining deception prevalence in autistic children, adolescents, and adults in a social setting. When required to lie face-to-face in a hide-and-seek paradigm, autistic individuals were less likely to attempt to lie compared with neurotypical individuals. Yirmiya et al. suggest that autistic individuals may have lied less frequently because they experienced difficulties with their ToM (indicated by their performance on an adapted Sally-Anne task). Although this study was situated in a social setting, it did not address whether autistic adults choose to lie of their own volition as participants were instructed to deceive. In the only study to date (to our knowledge) directly investigating autistic adults’ independent lying choices, Van Tiel et al. (2021) asked autistic and neurotypical adults (matched on age and IQ) to play a computer strategy game in which they could win a prize by choosing to deceive. Relative to neurotypical controls, autistic participants were just as likely to choose to deceive and learned patterns of deception at a quicker rate. Thus, counter to widely held beliefs, Tiel et al.’s results suggest that autistic adults can, and do, willingly engage in deceit. Recently, utilizing a self-report questionnaire, Bagnall et al. (2024) found that autistic and neurotypical adults (matched on IQ) were similarly likely to report that they would lie in everyday situations. However, the social demands experienced in naturalistic in-person interactions were not present in either Van Tiel’s computerized task or Bagnall’s questionnaire. As autism is defined by socio-communicative difficulties (American Psychiatric Association, 2013), autistic adults’ choices to engage in deception may differ when tested in face-to-face social situations. In particular, the social pressures experienced during face-to-face interactions are known to exacerbate autistic adults’ difficulties associated with information processing speed (Luke et al., 2012; Zapparrata et al., 2022). Therefore, it is crucial that future research investigates autistic adults’ lie telling behaviors in realistic social settings to increase the ecological validity of findings and their generalizability to everyday situations.

Currently, we can only speculate about why—or even if—autistic adults choose to lie less frequently than neurotypical adults in naturalistic social situations. Bagnall et al. (2024) propose that some autistic adults may lie as frequently as neurotypical adults in everyday situations, and suggested that lie inclination may be related to factors such as moral acceptability, self-rated lie-telling ability, and processing speed (e.g., executive functioning). Whereas other evidence suggests that autistic adults may choose to lie less frequently than neurotypical adults due to differences in their ToM (Yirmiya et al., 1996). If autistic adults produce fewer prosocial lies, this may place strain on their relationships and contribute to higher levels of loneliness (Grace et al., 2023). Hence, there is a pressing need for future research to examine autistic adults’ lying decisions in social settings, with a particular focus on identifying the conditions under which they do or do not engage in deception and—most importantly—why. If research identifies incongruencies between social expectations and autistic adults’ lie-telling decisions, training programs could be developed to teach autistic adults about prosocial lying for one’s own benefit or the benefit of others.

## Step 2: the communicator’s behavior provides cues to deception and honesty

Step 2 of the Brunswik Lens Model of Deception (Hartwig and Bond, 2011) proposes that once an individual has decided to lie, they will begin to display cues to deception through their behavior. Zuckerman et al. (1981) claimed that behavioral cues are indicative of psychological processes that individuals experience when lying and proposed four factors that predict deception cues: cognitive processing, behavioral control, affect, and arousal.

### Cognitive processing

The cognitive processing factor posits that telling a lie is more cognitively demanding than truth-telling as one must suppress the truth, fabricate details, and avoid logical inconsistencies (Zuckerman et al., 1981). The increased cognitive demand of these processes on executive functioning is believed to be reflected in liars’ non-verbal behaviors including their eye-movements (Mann et al., 2012), iconic-illustrators (hand gestures that supplement verbal information; DePaulo et al., 2003), verbal behaviors (e.g., details included in verbal responses; Luke, 2019), and response latency (time until response; DePaulo et al., 2003).

While neurotypical adults may sometimes struggle with the cognitive demand experienced when lying, it has been proposed that executive functioning limitations underpin many of the social and cognitive difficulties faced by autistic adults (Johnston et al., 2019). The executive dysfunction hypothesis (Pennington and Ozonoff, 1996) states that difficulties with executive functioning (including difficulties with working memory, inhibition of thought, and planning) may be responsible for a large proportion of behavioral difficulties in autism, including difficulties in ToM. According to the literature, over 40% of autistic individuals are believed to meet the diagnostic criteria for Attention-Deficit Hyperactive Disorder (inattentiveness, hyperactivity, impulsivity and emotional dysregulation; American Psychiatric Association, 2013; Joshi et al., 2013, 2017; Hours et al., 2022). Thus, autistic adults who naturally experience difficulties with cognitive processing may find lying even more demanding if they struggle to inhibit truthful responses or have difficultly holding lies in short-term memory. Such differences in cognitive processing may cause several well-studied deception cues to differ across autistic and neurotypical adult populations.

Firstly, neurotypical liars are stereotypically believed to avert their gaze due to feeling guilty or ashamed (DePaulo et al., 2003). However, research suggests that liars may actually attempt to make more eye-contact to counter this stereotype and appear credible (Mann et al., 2012). Mann et al. (2012) discovered that gaze aversion did not differ between liars and truth-tellers, however, liars made more deliberate eye-contact than truth-tellers. By contrast, Trevisan et al. (2017) report that autistic adults often avoid eye-contact to prevent sharing confidential information and guard against sensory overload. Therefore, autistic adults may conceivably conform with neurotypical stereotypes by displaying higher levels of gaze aversion when lying (beyond their natural level of ASD-related gaze aversion) in order to minimize processing information transmitted via eye-contact (reducing cognitive demand) and focus on maintaining their lie (Itier and Batty, 2009).

Secondly, neurotypical adults’ use of iconic illustrators (e.g., pointing left when saying “we went left”) decreases while producing deceptive communication (DePaulo et al., 2003). This behavior change may occur because neurotypical liars’ cognitive resources are restricted by the additional executive functioning demands associated with creating and maintaining a lie. Comparatively, autistic speakers may naturally produce more iconic gestures when communicating than neurotypical speakers as they lighten cognitive load by supplying visuospatial information (Medeiros and Winsler, 2014). Hence, autistic adults may produce more iconic illustrators than neurotypical adults when lying to display their point visually, reducing pressure on their cognitive resources. Alternatively, we speculate that the additional cognitive demand experienced when lying may reduce autistic adults’ use of iconic illustrators (similar to neurotypical adults) due to their cognitive resource availability. Further research is required before any strong conclusions can be drawn concerning autistic adults’ use of iconic-illustrators during deception.

Thirdly, length of time taken to answer questions (response latency) is believed to increase when neurotypical adults lie due to increased demands on executive functioning processes (Gombos, 2006). Although autistic adults may naturally take longer to respond verbally due to differences in information processing speed (Velikonja et al., 2019), they may take even longer to respond when telling lies because of executive functioning difficulties reducing their information processing capacity and restricting their working memory, inhibition, and planning (Hill, 2004). Due to the additional executive functioning difficulties that autistic adults may experience, the difference in response latencies between truthful and deceptive communication may be even greater in autistic adults compared to neurotypical adults.

Finally, due to the cognitive exertion required to successfully deceive, neurotypical liars tend to include fewer details in deceptive responses compared to truthful responses (Luke, 2019). Although the deception literature has highlighted the importance of response detail, it is yet to fully consider how cognitive processing biases influence this verbal behavior. While neurotypical adults usually process information globally (e.g., they see the whole before the parts), autistic adults often exhibit detail-focused local processing biases (e.g., they see the parts before the whole; see Happé (2005) for an account of the weak central coherence theory of autism). Consequently, autistic adults may include more specific detail in both truthful and deceptive responses relative to neurotypical adults. Only one study to date has examined details in deceptive responses; in Bagnall et al. (2023), neurotypical and autistic adults enacted a mock-crime and completed a face-to-face interview in which all participants were instructed to claim their innocence. They discovered that “innocent” and “guilty” autistic and neurotypical mock-suspects reported similar levels of investigation-relevant information.

### Attempted behavioral control

Zuckerman et al. (1981) argues that neurotypical adults attempt to control their behavior when they deceive to avoid arousing suspicion. For example, in order to appear credible, a liar may fail to correct their mistakes (DePaulo et al., 2003) or try to avoid producing self-manipulators (movements that occur against or on the body, such as playing with hair; Ekman and Friesen, 1972). An-as-yet-untested possibility is that some autistic adults’ efforts to control their non-verbal behavior during deception may be more successful and convincing due to their extensive experience of social camouflaging (also known as “masking”; Sedgewick et al., 2021).

Although there are disagreements concerning the prevalence of social camouflaging due to variations in behavior operationalization and measures between studies (Cook et al., 2021), most researchers agree that social camouflaging involves compensation (observing and copying others’ behavior), masking (dampening autistic characteristics by monitoring one’s own behavior and consciously presenting a non-autistic persona), and assimilation (using behavioral strategies to “fit in” with others; Alaghband-Rad et al., 2023). Social camouflaging can be so convincing that the use of these social imitation strategies can result in late or missed diagnosis (Kopp and Gillberg, 1992). In a recent online study, Cage and Troxell-Whitman (2019) discovered that over 70% of autistic adults reported they constantly engaged in social camouflaging. Self-reported levels of social camouflaging are also consistently higher in autistic females than autistic males (Hull et al., 2020), potentially due to differences in stigmatization between genders (Cage and Troxell-Whitman, 2019) and/or gender differences in underpinning cognitive abilities (Lehnhardt et al., 2016). Although motivations underpinning masking and deception are often distinct (e.g., mask to fit in with neurotypical friends vs. lying to protect them), this sophisticated and practiced ability to alter one’s behavior could, in theory, enhance an autistic adult’s ability to control their presentation of deception cues. For example, it is possible that autistic females who have developed advanced social camouflaging skills may display fewer non-verbal cues to deceit than autistic males and neurotypical adults. However, some autistic adults may be unaware of their masking if it is a subconscious defense mechanism utilized to avoid feeling isolated or marginalized during social interactions (Pearson and Rose, 2021). Consequently, some autistic adults who mask frequently may still struggle to control their non-verbal cues while engaging in deception.

Self-manipulators bring comfort to individuals during stressful situations. Despite their soothing nature, evidence suggests that on average most neurotypical adults tend not to produce more self-manipulators when lying, potentially to avoid appearing nervous and arousing suspicion (DePaulo et al., 2003; Porter et al., 2008; Porter and ten Brinke, 2010). If autistic adults are unaware that self-manipulators arouse suspicion due to differences in understanding others’ perspectives (Baron-Cohen, 2008), they may not consciously control these behaviors while lying and may produce these behaviors more frequently. However, if autistic adults are aware that self-manipulators arouse suspicion, they may be able control these behaviors more successfully than neurotypical adults due to their social camouflaging experience. Depending on individual differences, both predictions may be accurate with some autistic adults producing fewer self-manipulators when lying while others may produce more. Further research is required to investigate these predictions regarding the frequency of self-manipulators produced by autistic adults while engaging in deception.

Analyses of verbal responding show that neurotypical liars are less likely to correct their mistakes than truth-tellers (DePaulo et al., 2003). This is because many liars have stereotypical beliefs about the appearance of truthful responses (e.g., do not include mistakes or self-doubt) and so alter their deceptive responses to align with these beliefs. Some studies suggest that neurotypical children produce fewer self-corrections when lying (Saykaly et al., 2013; Talwar et al., 2018), while other studies involving adults suggest the rate of self-corrections may increase (Lee et al., 2008). Therefore, additional research is required to support confident conclusions concerning neurotypical adults’ self-corrections when lying. The frequency of autistic adults’ self-corrections during deception may vary due to differences in their social understanding and awareness of how their language may influence others’ beliefs (Lake et al., 2011). Individuals correct themselves if they detect that their mistake incorrectly altered their conversation partner’s belief—a process contingent on ToM. Autistic adults who experience ToM difficulties may not recognize how their mistakes inaccurately influence their communication partner’s beliefs (e.g., they may not realize that their beliefs differ from their partner’s) and therefore see no need to correct themselves. Consequently, autistic adults may make fewer self-corrections than neurotypical adults both when truth-telling and lying.

### Affect

Adverse emotions experienced while lying, such as guilt, may influence various aspects of behavior including expression of affect, frequency of self-manipulators, and vocal pitch (Zuckerman et al., 1981; Villar et al., 2013). If experiencing certain emotions predicts the occurrence of particular deception cues, we may expect the appearance of deception to differ in autistic adults who experience heightened emotional responses (Samson et al., 2015) or difficulties with emotional control (Mazefsky et al., 2013) compared to other autistic and neurotypical adults who do not experience such emotional responses. While neurotypical adults may not increase their level of self-manipulation when lying, autistic adults may do so due to the elevated levels of anxiety they are reported to experience during deception (Grandin, 2006). As self-manipulators serve a self-soothing function (analogous to self-stimulatory behaviors that are common in autism, known as stims; Lawson et al., 2014), autistic adults may produce these behaviors more frequently while engaging in deception as a means of reducing stress.

Additionally, heightened emotions experienced while lying are believed to increase tension in the vocal tract, increasing pitch. Villar et al. (2013) reported that participants produced an average pitch frequency of 203.38 Hz during truthful responses and 217.17 Hz during deceptive responses. Compared to neurotypical individuals, autistic individuals have increased pitch variability (Bonneh et al., 2011) and are more likely to produce slow or extremely fast speech (Baron-Cohen and Staunton, 1994). Like neurotypical adults, autistic adults average pitch frequency may increase when lying due to heightened anxiety tightening their vocal tract. However, these remain as-yet-untested hypotheses. With the rise of the application of machine learning in deception detection (Otasowie, 2020), it is important to examine differences in pitch variation—above and beyond what is natural in autistic populations—to ensure that autistic adults are not erroneously judged as deceptive.

### Generalized arousal

Zuckerman et al. (1981) proposed that when an individual lies, their autonomic nervous system’s responses cannot be consciously controlled, leading to unconscious behavior changes including pupil dilation and elevated heart rate (DePaulo et al., 2003). As a result, physiological methods—such as polygraphs—have been developed to detect deception by measuring electrodermal activity (Grubin and Madsen, 2005). Autism has been linked with atypicalities in the anterior cingulate cortex (Fan et al., 2012) and amygdala (Nordahl et al., 2012), brain structures responsible for modulating autonomic responses. Additionally, research has identified autonomic dysregulation in some autistic children, including elevated resting heartrates (Ming et al., 2005) and amygdala hypoactivation (Herrington et al., 2016). This autonomic dysregulation may skew results if autistic adults were to complete a physiological measure of deception detection (e.g., a polygraph), potentially causing them to be erroneously judged as deceptive. Although polygraph evidence is not permittable in UK courts, the police and probation service do utilize polygraphs to assist investigations, monitor risk, and assess parole conditions (Home Office, 2019). Future research should consider investigating the autonomic responses that autistic adults experience when lying to ascertain whether standard physiological deception-detection methods can be reliably and ethically applied to autistic adults.

In summary, it is vital that research investigates the appearance of deceptive communication in autism as the natural characteristics of this condition can map directly on to stereotypical deception cues (e.g., averted eye-gaze). As a result, autistic adults may be unfairly judged against expectations based on neurotypical behaviors, leading to inaccurate judgments of deceit. Such misinterpretations of behavior could have negative consequences for autistic individuals, especially in forensic contexts such as courtrooms. Blackhurst et al. (2022) discovered that a defendant displaying common characteristics of autism (e.g., averted eye-gaze) was initially judged to be blameworthy and deceptive until participants were informed that they were autistic. Following disclosure of the defendant’s diagnosis, judgments of blame and honesty, respectively, decreased and increased. However, there are currently no published papers investigating how autistic adults produce deception cues or investigating the psychological mechanisms which underpin such cues in this population. To reduce the likelihood of inaccurate veracity judgments due to misinterpretations of behavior, investigating the characteristics of truthful and deceptive communication in autistic adults represents an important objective for the autism research community. We summarize our theory-driven and evidence-based predictions concerning autistic adults’ production of deceptive behaviors in Table 1.

**Table 1:** Summary of the literature investigating deceptive behavior in neurotypical adults (NT), how autism influenced behavior (ASD), and possible hypotheses concerning how these behaviors may manifest in autistic liars.

Behavior	Effect of ASD	Effect of deception in NT adults	Possible hypothesis for ASD
**Non-verbal**			
Eye-contact	Decreased eye-contact (American Psychiatric Association, 2013)	Liars > Truth-tellers (Mann et al., 2012)	Liars < Truth-tellers
Self-manipulators	Increased self-manipulators in the form of stimming (Kapp et al., 2019)	Liars = Truth-tellers (Porter and ten Brinke, 2010)	Liars >/< Truth-tellers
Iconic illustrators	Use more iconic illustrators than other hand gestures (Medeiros and Winsler, 2014)	Liars < Truth-tellers (DePaulo et al., 2003)	Liars >/< Truth-tellers
**(Para) verbal**			
Pitch	Unusual and variable pitch (McCann and Peppé, 2010)	Liars > Truth-tellers (Villar et al., 2013)	Liars > Truth-tellers
Response latency	Increased response latencies due to reduced information processing capacity (Hill, 2004)	Liars > Truth-tellers (DePaulo et al., 2003)	Liars > Truth-tellers
Details in response	Detailed-focus processing (Booth and Happé, 2010)	Liars < Truth-tellers (Taylor et al., 2017)	Liars = Truth-tellers
Self-corrections	Differences in social understanding and ToM make autistic adults less likely to recognize the need for self-corrections (Lake et al., 2011)	Liars < Truth-tellers (DePaulo et al., 2003)	Liars < Truth-tellers

## Step 3: judge perceives behavioral cues

Step 3 of the Brunswik Lens model’s application to deception captures how a judge may perceive a liar’s cues. Crucially, verbal, and nonverbal cues stereotypically perceived to be indicative of deception are not necessarily reliable deception cues (Feeley and Young, 2000). There is unanimous agreement among researchers that the general population and legal professionals uphold, and act on, inaccurate cues to deceit (Strömwall and Granhag, 2003; Vrij, 2008). For example, many people incorrectly believe averted eye-gaze and fidgeting to be indicators of deceit (Bogaard et al., 2016). Such stereotypical deception cues overlap with natural characteristics of autism, meaning that autistic adults are more likely to avert their gaze and fidget even when telling the truth (American Psychiatric Association, 2013). Misconceptions regarding deception cues, in conjunction with limited knowledge concerning natural autistic behaviors, could lead neurotypical observers to erroneously judge autistic adults as deceptive in everyday and forensic situations (Logos et al., 2021).

DePaulo et al.’s (2003) meta-analysis of deception cues did not support popular stereotypical beliefs, instead discovering only small-to-moderate associations between deception and the following cues: pupil dilation, negative facial expressions, fewer hand movements, increased fidgeting, vocal pitch, less coherent responses, shorter response lengths, fewer details, and avoidance of imperfections. Luke (2019) used data simulations to show that many effect sizes for deception cues, including those identified by DePaulo et al., are greatly inflated by low power and small numbers of estimates. Overreliance on inaccurate deception cues, and small effect sizes associated with more “reliable” cues, may explain how perception of behavior changes could negatively influence veracity decisions.

The Global Deception Research Team (2006) conducted a comprehensive investigation of deception beliefs, sampling 2,320 neurotypical adults across 58 countries. Over 60% of their respondents believed that gaze-aversion is a deception cue, and over 20% stated that body movements and incoherent speech are also indicative of deceit. Hurley et al. (2014) reported similar results in their sample of 161 neurotypical university students, but also investigated the origins of these beliefs. They discovered that the most frequent origin of beliefs about deception was observed behavior (52% of the sample), with mass media and personal experience also mentioned relatively frequently (around 20% each). These data suggest that inaccurate beliefs concerning deception cues are transmitted at both macro (observed behavior) and micro-levels (individual experiences). These findings align with developmental theories proposing that neurotypical children learn how to deceive, and what deception looks like, through social interactions and behavior modeling (Talwar and Crossman, 2011; Engarhos et al., 2020). However, social constructivist theory argues that autistic children spend less time engaged in communicative interactions than neurotypical peers, reducing their opportunities to make social connections or to model behavior (Bauminger et al., 2003; Goddard and Cook, 2022). This reduction in social experience, coupled with the fact that autistic children may not innately or spontaneously orientate toward social stimuli (Banire et al., 2020), may limit opportunities to learn and observe deceptive behavior. If autistic children do not prioritize social information, they may be less likely to pay attention to others’ behavior and thus fail to identify both actual and perceived deception cues. As such, autistic adults may potentially be more likely to rely on micro-level sources (e.g., individual experiences) to guide their perception of deception cues compared to macro-level sources. However, autistic children who are relatively more social will have increased opportunities to observe and model social behaviors (including deceptive behaviors) at the macro-level, which may potentially facilitate their engagement in effective social camouflaging as an adult (Hull et al., 2017; Cook et al., 2021). Autistic individuals who display sophisticated camouflaging skills may be more perceptive of deceptive cues than those who did not engage in social interactions as frequently during childhood, and/or experience reduced capability or personal desire to mask as an adult.

If autistic adults do not attend to stereotypical non-verbal deception cues, this could conceivably free up cognitive resources to attend to more diagnostic cues to deceit, such as paraverbal cues. As individuals who focus on paraverbal deception cues are more accurate lie detectors than those who focus on non-verbal cues (DePaulo et al., 1982), an autistic adult’s reduced preference for observing others’ behavior could conceivably improve their lie detection accuracy if they were able to focus on auditory information and made aware which paraverbal cues are indicative of deceit. Evidence suggests that some autistic individuals experience heightened auditory sensitivity and discriminatory abilities, including pitch discrimination/memory (Heaton et al., 2008). Such abilities could make autistic adults more efficient at perceiving paraverbal cues to deceit compared to neurotypical adults, which may increase their deception detection accuracy. However, not all autistic adults experience heightened auditory sensitivity with many experiencing some degree of sensory dysfunction related to processing auditory, visual, or vestibular stimuli (Robertson and Baron-Cohen, 2017). Some autistic adults experience sensory issues that reduce their listening abilities in social situations (e.g., central auditory processing disorder; Ocak et al., 2018). Consequently, autistic adults’ deception detection accuracy may decrease if difficulties in processing auditory information impacts their ability to perceive paraverbal cues. It is important for future research to identify factors that determine whether autistic adults can perceive paraverbal deception cues as this ability is integral to making accurate veracity decisions.

Whether autistic adults are sensitive to non-verbal and/or paraverbal deception cues is currently an open question. Decreased sensitivity to all cue types could potentially explain why autistic adults are more likely than neurotypical adults to fall victim to certain types of fraudulent crime as they fail to notice or encode signs of deceit (Action Fraud, 2019). However, as autistic adults can be highly perceptive when processing visual and auditory stimuli (Heaton et al., 2008; Mottron et al., 2013), it may be possible to enhance their detection of particular behaviors that are reliably considered to be diagnostic of deceit through training (DePaulo et al., 2003). Legal professionals have recently begun to focus more heavily on verbal cues to deception (e.g., details and self-corrections), with the introduction of criterion-based content analysis enabling police to distinguish the veracity of statements more successfully (Vrij et al., 2018). Training programs focusing on the identification of verbal deception cues could potentially be adapted to enhance autistic adults’ lie detection abilities and provide them with the skills to protect themselves against manipulation.

In addition to affecting how autistic adults perceive deception cues, autism may also influence how they are perceived by others. In Lim et al. (2022), 30 autistic and 29 neurotypical adults filmed video interviews before 1,410 neurotypical adults rated the interviewees’ credibility and truthfulness. They found that autistic adults were perceived to be more deceptive than neurotypical adults, even when telling the truth. Although Lim et al. did not link these results to specific characteristics of autism, they concluded that an autistic adult’s overall behavioral presentation may lead to erroneous veracity decisions. This effect could be explained by Expectancy Violation Theory, which argues that people anticipate others to behave in certain ways during social interactions (Burgoon, 2015). If these expectations are violated (e.g., a communication partner averts their gaze), the individual’s attention is drawn to unexpected behaviors and they will seek an explanation (e.g., a communication partner is continually averting their gaze because they are lying). However, awareness that a communication partner has autism may modify expectations and alter how certain behaviors are perceived, leading to more positive judgments (Blackhurst et al., 2022). In order to educate people on the danger of inaccurate perceptions, it is crucial for future research to identify if any **specific** characteristics of autism are misconstrued as deceptive and examine actual cues to deceit in autistic adults (if any exist).

To conclude, both neurotypical and autistic adults are likely to harbor inaccurate beliefs about deception cues due to deep-rooted social stereotypes regarding the appearance of liars (Global Deception Research Team, 2006). However, due to differences in social experiences (Goddard and Cook, 2022) and sensory processing (Robertson and Baron-Cohen, 2017), autistic adults may perceive deception cues differently than neurotypical adults—although whether these differences increase or decrease deception detection accuracy is currently unknown and requires further investigation. If future research could identify a psychological mechanism (e.g., sensory processing, lack of an innate orientation toward social stimuli) that underlies differences in autistic adults’ perception of deception cues, this could inform the development of new educational or anti-victimization programs designed to improve autistic adults’ deception detection.

## Step 4: making the veracity decision

The final step in applying the Brunswik Lens Model to deception addresses how the judge incorporates perceived cues into their veracity decisions. While many people naively believe themselves to be adequate lie detectors, DePaulo et al. (2003) meta-analysis showed that average accuracy for lie detection among neurotypical adults is 54% (when 50% represents chance accuracy). There are numerous explanations for why neurotypical individuals’ veracity decisions are so inaccurate. The truth-default theory (TDT; Levine, 2014) proposes that humans are naturally truth-biased; we tend to believe people only communicate things that are true based on Grice’s (1989) conversational maxim of quality. For the truth-default to be abandoned, trigger events—such as perceiving deception cues—must be experienced by the recipient of communication. If a trigger is sufficiently strong (e.g., multiple deception cues are perceived), suspicion is generated, and the truth-default may be temporarily discarded while the message’s veracity is scrutinized. If the threshold for suspected deception is not met, truth-bias returns and the message is judged as honest. If the deception threshold is met, the truth-default is abandoned, and the message is judged to be deceptive.

Although TDT (Levine, 2014) proposes that perception of deception cues can lead to judgments of deceit, this process may be erroneous due to lie-detectors’ overreliance on unreliable cues (Vrij, 2008). It is well documented that lie-detectors are heavily dependent on non-verbal deception cues (e.g., averted eye-gaze; Global Deception Research Team, 2006) even though deceit cannot be reliably inferred from these (DePaulo et al., 2003). Fixation on stereotypical non-verbal cues may lead to a “lie-bias” whereby people falsely believe people to be deceptive (Global Deception Research Team, 2006). By contrast, Bond and DePaulo’s (2006) meta-analysis reported that lie-detectors performed more accurately when they could only hear a communicator (63% accuracy) versus being able to see and hear them (52% accuracy). Therefore, veracity decisions primarily informed by verbal and paraverbal deception cues may have increased diagnostic accuracy.

While Levine (2014) claims the truth-default to be universal, it is possible that this state may vary across neurodiverse populations and the extent to which autistic adults display a truth-bias is currently unclear. On one hand, over 40% of autistic adults experience co-occurring anxiety disorders (Zaboski and Storch, 2018), potentially increasing their threat sensitivity due to hypervigilance and attentional biases for threat-related stimuli lowering their truth-bias threshold (Mogg et al., 2000). On the other hand, differences in communication skills and decreased orientation to social stimuli (Banire et al., 2020) may reduce the likelihood that autistic adults notice deception cues that would trigger their truth-bias threshold. Furthermore, some autistic adults experience increased social naivety and are more vulnerable than neurotypical adults and individuals with other intellectual disabilities to victimization in the form of bullying, sexual abuse, and physical abuse (Mandell et al., 2005). It is possible that reduced suspicion of deceit during social interactions, social naivety, and difficulties detecting the intentions of others, could contribute to this vulnerability by increasing truth-bias. A greater understanding of truth bias could help develop interventions to reduce social vulnerability experienced by some autistic adults.

To date, only Williams et al. (2018) have directly examined lie detection ability in autistic adults. Neurotypical and autistic adults (matched on IQ and age) completed the Autism Quotient (50 statement-version to measure autistic traits) and a lie detection task. Their results showed that autistic adults’ veracity decisions were significantly less accurate (46% accuracy) than neurotypical adults’ (72% accuracy). Autistic adults struggled to make deceptive inferences even when deception cues were explicit, suggesting that they may fail to recognize or incorporate deception cues into their veracity decisions. This finding may suggest that autistic adults have a stronger truth-bias compared to neurotypical controls. Indeed, as Williams et al. presented lies to participants in videos, differences between autistic and neurotypical adults’ veracity decision accuracy may be greater under naturalistic conditions that increase demands on social, mentalizing, and information processing systems.

It is important to consider how individual differences in cognition and information processing systems may influence sensitivity to deception cues when making veracity decisions. Executive functioning processes—including working memory and metacognitive awareness—are required at each stage of the veracity decision (Weber, 2016). Judges must identify cues to deception, hold this knowledge in their short-term memory, and then integrate this information into their veracity decisions alongside competing information. However, many autistic adults experience difficulties with executive functioning (including working memory, flexibility of thought, and attentional inhibition; Hill, 2004) that may decrease their ability to detect deception. For example, some autistic adults may struggle to pull information about observed deception cues into their veracity decision, while also considering competing information. There is currently little research focusing on the influence of executive function on deception detection in autistic adults, with only one study proposing that high executive load may offer a possible explanation for why autistic adults may struggle to detect deceit in certain circumstances (Williams et al., 2018). Therefore, further research is required to strengthen such claims relating to the impact of executive functioning on deception. Identifying an aspect of executive dysfunction linked to reduced deception detection accuracy could potentially inform interventions designed to promote lie detection capabilities in autistic adults.

Crucially, the veracity decision making process is only initiated once the judge suspects a communicator may be lying. This consideration requires ToM, as the judge must recognize the possibility that a communicator may be attempting to implant a false belief (Lee, 2013). Autistic adults who experience difficulties with ToM may be relatively less likely to clear the initial hurdle of suspecting deceptive intentions, despite being able to perceive behavioral differences between deceptive and truthful communication. Williams et al. (2018) investigated the link between ToM, autistic traits, and lie detection in neurotypical adults. Their results revealed that participants with more autistic traits were significantly less accurate at detecting deception, but this effect was not mediated by ToM. However, this study did not include participants with formal autism diagnoses and their performance on ToM tasks fell within the normal range for neurotypical development, restricting the generalisability of these findings to autistic adults. Predictive relationships between ToM and deception in diagnosed autistic adults require further investigation.

Finally, it is important to note that there may be some autistic adults who can detect lies just as well, if not better, than neurotypical adults. Not all autistic adults experience difficulties with their executive functioning or ToM (American Psychiatric Association, 2013; Jones et al., 2018), and such individuals may not experience as pronounced difficulties with their lie detection ability. Furthermore, some autistic adults display hyper-attention to detail and sensory hypersensitivity, making them extremely talented at input-operation-output reasoning (If X, consider Y, conclude Z; Baron-Cohen et al., 2009). If an autistic adult knew which cues to deception were the most reliable indicators of deceit, they could perhaps use this reasoning to assist with deception detection.

Whether and/or why autistic adults struggle to detect deception remains unclear. Based on current understanding of the autism spectrum, we predict that autistic individuals who face difficulties with their ToM, executive functioning, and social communication may make more inaccurate veracity decisions. It is essential that research begins to investigate lie detection in autistic adults, as weaknesses associated with this process could significantly increase their susceptibility to manipulation, coercion, and fraudulent crime.

## Conclusion

The present theoretical review has demonstrated how the Brunswik Lens Model (Hartwig and Bond, 2011) could provide valuable and comprehensive theoretical direction to the study of deception in autistic adults. By adopting this model, future research can directly target how individual mechanisms—and relationships between them—differ between autistic and neurotypical adults. Organizing research in relation to the model will allow researchers to systematically test theory-driven predictions regarding how autism may influence autistic adults’ production, awareness, and detection of deception. Working from a joint theoretical perspective can inform the development of a comprehensive model of deception in autism and interventions to help protect autistic adults against manipulation and victimization.

Applying a unified theoretical model is required to broaden the scope of research investigating deception in autism, thereby addressing a key weakness of the existing literature. The vast majority of research to date has focused on stages 1 and 4 of the Brunswik Lens Model (lying prevalence and deception detection; Williams et al., 2018; Van Tiel et al., 2021), while largely neglecting stages 2 and 3 (production and perception of deception cues) and overlooking the psychological mechanisms which may influence these stages. Investigating stages 2 and 3 is necessary to advance understanding of how autistic adults engage in deception, how they perceive others’ deception cues, and how their deception cues are perceived by others. Throughout this review we have highlighted how ToM, executive functioning, and social experience may individually or interactively influence all four stages of the Brunswik Lens Model. Comprehensive investigation of causal mechanisms is necessary to provide crucial information about the inner processes of deception and explain differences in deceptive-decision making between autistic and neurotypical adults.

Moreover, understanding of stages 2 and 3 will enable researchers to address prominent issues faced by autistic adults, such as the potential to be erroneously judged as deceptive based upon misinterpretation of their naturalistic behavior. Studies investigating how autistic adults perceive deception are required to inform the development of social training and educational programs designed to ease social interactions and protect individuals with heightened vulnerability (e.g., anti-victimization interventions). Ultimately, comprehensive investigation of stages 1–4 will lay the foundation for the development of a unified-system theory of how autism influences the Brunswik Lens Model of deception with important benefits for both knowledge and practice.

In addition to discovering whether and how autistic adults differ at each stage of the Brunswik Lens Model compared to neurotypical adults, it is important to consider why they differ. Using the Brunswik Lens Model, researchers may be able to identify specific difficulties related to the deception process in autism and work toward situating these within the broader profile of social-communication difficulties that characterize the condition. Identifying the origins of deception difficulties in relation to an individual’s broader autism profile, and potential differences across the autistic population, may inform the design of interventions designed to strengthen core socio-communicative skills in autistic adults and, in turn, their veracity decision making.

Despite a recent influx of publications investigating deception in autism, most studies focus on autistic children or adolescents (Bagnall et al., 2022). As autism is a life-long condition, and the consequences of difficulties producing and/or detecting deception may increase with age, future research should directly target adults. Difficulties telling lies could negatively impact autistic adults’ maintenance of satisfying social relationships (Henderson et al., 2023), leading to feelings of isolation and poor mental health (Schiltz et al., 2021). Reduced lie detection accuracy may increase autistic adults’ risk of victimization and vulnerability to manipulation, heightening their risk of falling victim to crimes such as fraud (Action Fraud, 2019). If autistic adults fall victim to a crime, they may experience a police interview/trial in which their veracity may be questioned. It is also important that future research investigates how neurotypical individuals perceive autistic adults’ behavior and whether misconceptions regarding natural autistic behaviors contribute to inaccurate veracity judgments. As such, there is a pressing need to investigate stages 1–4 of the Brunswik Lens Model in order to yield insight into, and consider ways of alleviating, the negative consequences autistic adults may face throughout the deceptive-decision making process.

To strengthen the field going forward, it is necessary to address the weaknesses of extant research. For example, many deception studies use game-play deception methods in which individuals are instructed to lie to a computer. Computerized games afford high levels of environmental control, but lack ecological validity as they fail to convey the social demands experienced in everyday life. As autism is a condition characterized by social difficulties (American Psychiatric Association, 2013), future research should design naturalistic experiments in which participants must lie face-to-face to generate realistic data representing everyday social situations. Although practicality must be considered, future research would also benefit from greater sample sizes in order to find meaningful effects and counterbalance the historically small effect sizes associated with deception literature (Luke, 2019). Finally, almost nothing is formally known about the extent to which the autism community (autistic individuals, caregivers, clinical practitioners, etc.) is engaged with deception research including co-conceptualization, research design, and interpretation of data (Pellicano et al., 2014). To date, only one study (to the best of our knowledge) directly investigating deception detection in autistic adults has involved members of the autism community (Williams et al., 2018). Therefore, certain conclusions are being drawn about how autistic adults engage in deceptive communication without input from autistic adults themselves. Throughout this review we have provided alternative hypotheses to represent the heterogeneity of the autism spectrum and to reflect the current strength-based approach that is favored by many members of the autistic community (Lee et al., 2024). Moving forward, it is important to pursue community involvement to ensure studies investigating deception in autism have high internal validity and can be generalized to the realities of an autistic adult’s everyday life. Additionally, inviting autistic adults to input their opinions relating to research design may help guarantee that future research is conducted *with* the autistic community as opposed to *on* or *for* them (Pellicano et al., 2014).

To conclude, we encourage future researchers to consider utilizing the Brunswik Lens Model of Deception (Hartwig and Bond, 2011) to create a more comprehensive literature base, united in theory, relating to how autistic adults experience deceptive communication. This approach will significantly advance understanding of how autistic adults engage in deceptive communication in their everyday lives. With greater involvement of autistic participants, broader coverage of the entire deception process (from stage 1 to 4), and more ecologically valid methods, the autism and deception literature can overcome current methodological flaws to produce results with increased validity and generalisability. Finally, the creation of a complete model of deception in autism that encapsulates psychological mechanisms underpinning differences at each stage may have numerous practical implications, including informing the development of evidence-based interventions and educational programs to help safeguard future outcomes for autistic adults. Implementing the Brunswik Lens Model of Deception Detection in future research will allow the field to make substantive progress toward achieving an inclusive understanding of how autistic adults produce, perceive, and detect deceptive communication.

## Author contributions

TB: Conceptualization, Writing – original draft, Writing – review & editing. LW: Supervision, Writing – review & editing, Conceptualization. AR: Writing – review & editing. CH: Supervision, Writing – review & editing, Conceptualization.
